# Dr. Marshall H. Webb

**Published:** 1883-01

**Authors:** 


### OBITUARY.

*Dr. Marshall H. Webb.*—A few days ago we learned of the death of one who, although comparatively young in years, had already gained for himself an enviable reputation as a dental practitioner, and who occupied the responsible position of Lecturer on Operative Dentistry in the Dental Department of the University of Pennsylvania. Dr. Webbs' name is familar to all the progressive members of the profession throughout the country, as he was not only an accomplished operator, but a faithful contributor to the journals of our specialty. Highly cultivated in the science and art of dentistry, he had acquired that dexterity of hand so necessary to perform the nice and delicate as well as the most difficult operations on the teeth and to such a degree as to give him the reputation of being one of the most accomplished operators of the present time. We fear that Dr. Webb in his noble aspirations to accomplish his professional mission, neglected his material nature, until it began to fail, totter, and sink under injudicious burdens, though heaped upon it with the purest motives. A small force will often suffice to shatter in pieces earthen vessels, although they may contain the richest treasures. Thus genius departs, and the accomplished and admired are taken hence, while the green tendrils of friendship and love have scarce had time to twine fondly round their object.

Dr. Webb was a native of Lancaster County, Pennsylvania, and after graduating in the Philadelphia Dental College, located in Lancaster City, practicing also at stated times in Philadelphia. With a number of other American dentists, he visited the International Medical Congress held in London, England, in 1881, and by his participation in the proceedings of the Dental Sections and by his Clinics, he made a most favorable impression. His death is indeed a loss to the dental profession.

---

